# (Amino­acetato-κ^2^ *O*,*N*)bis­(quinolin-8-olato-κ^2^ *O*,*N*)cobalt(III) methanol solvate

**DOI:** 10.1107/S1600536808013135

**Published:** 2008-05-10

**Authors:** Bu-Qin Jing, Shuang-Ming Meng, Jing Han, Bin Wang, Xue-Mei Li

**Affiliations:** a College of Chemistry and Chemical Engineering, Shanxi Datong University, Datong, Shanxi 037009, People’s Republic of China

## Abstract

In the crystal structure of the title compound, [Co(C_2_H_4_NO_2_)(C_9_H_6_NO)_2_]·CH_3_OH, the Co^III^ atom is chelated by two quinolin-8-olate and one glycinate anions in a distorted octa­hedral coordination geometry. The five-membered chelating glycinate ring assumes an envelope conformation. The complex mol­ecules are assembled by inter­molecular N—H⋯O hydrogen bonding.

## Related literature

For a related structure, see: Li *et al.* (2003 ▶).
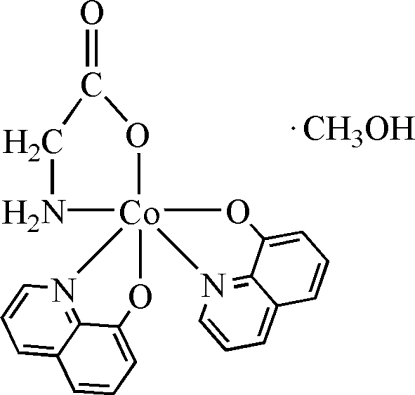


## Experimental

### 

#### Crystal data

[Co(C_2_H_4_NO_2_)(C_9_H_6_NO)_2_]·CH_4_O
*M* *_r_* = 453.33
Triclinic, 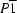

*a* = 9.8377 (4) Å
*b* = 10.6526 (4) Å
*c* = 10.7369 (4) Å
α = 82.047 (1)°
β = 76.289 (1)°
γ = 64.941 (1)°
*V* = 989.32 (7) Å^3^
*Z* = 2
Mo *K*α radiation
μ = 0.91 mm^−1^
*T* = 273 (2) K
0.20 × 0.15 × 0.12 mm

#### Data collection

Bruker SMART CCD area-detector diffractometer
Absorption correction: multi-scan (*SADABS*; Sheldrick, 1996 ▶) *T*_min_ = 0.840, *T*_max_ = 0.899
11346 measured reflections
3486 independent reflections
3261 reflections with *I* > 2σ(*I*)
*R*_int_ = 0.017

#### Refinement

*R*[*F* ^2^ > 2σ(*F* ^2^)] = 0.028
*wR*(*F* ^2^) = 0.093
*S* = 1.00
3486 reflections
281 parameters
H atoms treated by a mixture of independent and constrained refinement
Δρ_max_ = 0.28 e Å^−3^
Δρ_min_ = −0.36 e Å^−3^

### 

Data collection: *SMART* (Siemens, 1996 ▶); cell refinement: *SAINT* (Siemens, 1996 ▶); data reduction: *SAINT*; program(s) used to solve structure: *SHELXTL* (Sheldrick, 2008 ▶); program(s) used to refine structure: *SHELXTL*; molecular graphics: *SHELXTL*; software used to prepare material for publication: *SHELXTL*.

## Supplementary Material

Crystal structure: contains datablocks I, global. DOI: 10.1107/S1600536808013135/xu2418sup1.cif

Structure factors: contains datablocks I. DOI: 10.1107/S1600536808013135/xu2418Isup2.hkl

Additional supplementary materials:  crystallographic information; 3D view; checkCIF report

**Fig. 3.**
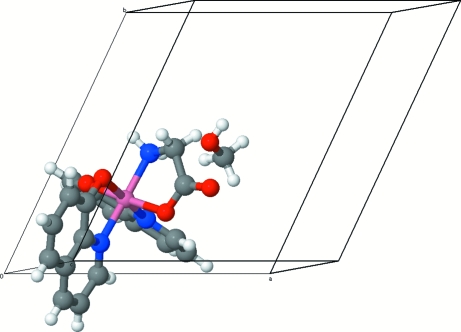
Interactive view of the complex.

Enhanced figure: interactive version of Fig. 3

## Figures and Tables

**Table:** 

Co1—O1	1.9045 (12)
Co1—O3	1.8926 (13)
Co1—O4	1.9002 (13)
Co1—N1	1.9373 (14)
Co1—N2	1.9179 (15)
Co1—N3	1.9309 (15)

**Table:** 

O3—Co1—O4	90.60 (6)
O3—Co1—O1	89.93 (6)
O4—Co1—O1	176.81 (5)
O3—Co1—N2	176.47 (5)
O4—Co1—N2	85.88 (6)
O1—Co1—N2	93.56 (6)
O3—Co1—N3	87.07 (7)
O4—Co1—N3	91.14 (6)
O1—Co1—N3	85.75 (6)
N2—Co1—N3	92.71 (7)
O3—Co1—N1	85.82 (6)
O4—Co1—N1	92.39 (6)
O1—Co1—N1	90.79 (6)
N2—Co1—N1	94.60 (6)
N3—Co1—N1	172.09 (7)

**Table 2:** Hydrogen-bond geometry (Å, °)

*D*—H⋯*A*	*D*—H	H⋯*A*	*D*⋯*A*	*D*—H⋯*A*
O5—H5⋯O2^i^	0.82	1.92	2.743 (3)	175
N3—H21⋯O5	0.85 (2)	2.17 (3)	2.950 (3)	153 (2)
N3—H22⋯O3^ii^	0.86 (3)	2.10 (3)	2.952 (2)	169 (2)

Symmetry codes: (i) 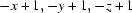; (ii) 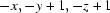.

**Fig. 1.**
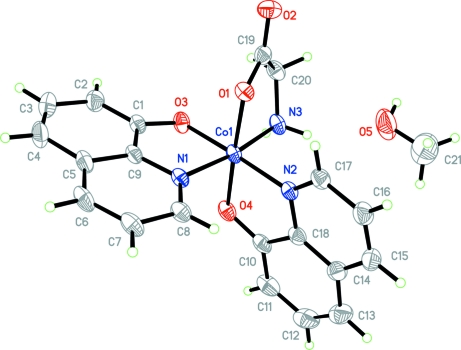
The atomic labeling scheme of (I) with displacement ellipsoids at the 30% probability level.

**Fig. 2.**
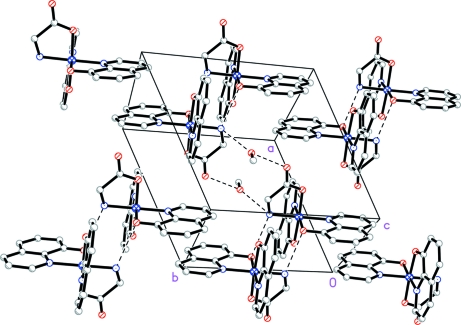
A packing diagram of the unit cell showing hydrogen bonds as dashed lines. H atoms, except for those involved in hydrogen bonds, are not included.

## supplementary crystallographic information

### Comment

The 8–hydroxyquinoline (HQ) is a very good ligand, forms complex compounds with various metal ions in solution. The strong chelating action of HQ in solution has been extensively studied and widely used in analytical chemistry. In this work, we use glycin and 8–hydroxyquinoline as bidedtate ligand to synthesize the title complex, (I).

The molecular structure of the title compound is shown in Fig. 1. The Co^III^ atom is chelated by two 8-hydroxyquinoline and one glycin anions in a distorted octahedral coordination geometry. The Co—N bond distances are longer than Co—O bond distances (Table 1), which agrees with that found in a related structure, tris(8-quinolinolato)-cobalt(III) methanol solvate (Li *et al.* 2003). The two 8-hydroxyquinolate rings are almost perpendicular to each other with a dihedral angle of 81.0°. The five-membered chelating ring of the glycin assumes an envelope conformation, with N3 atom at the flap position. The complex molecules are assembled by intermolecular N–H···O hydrogen bonding (Table 2). Lattice methanol molecule is linked with complex *via* O—H···O and N—H···O hydrogen bonding (Fig. 2).

### Refinement

Amino H atoms were located in a difference Fourier map and refined isotropically. Other H atoms were placed in calculated positions and allowed to ride on their attached atoms, with C—H = 0.93–0.97 Å, O–H = 0.82 Å, *U*_iso_(H) = 1.2 or 1.5*U*_eq_(C,*O*).

### Crystal data

**Table:**

[Co(C_2_H_4_NO_2_)(C_9_H_6_NO)_2_]·CH_4_O	*Z* = 2
*M**_r_* = 453.33	*F*_000_ = 468
Triclinic, *P*1	*D*_x_ = 1.522 Mg m^−^^3^
Hall symbol: -P 1	Mo *K*α radiation λ = 0.71073 Å
*a* = 9.8377 (4) Å	Cell parameters from 7882 reflections
*b* = 10.6526 (4) Å	θ = 2.4–28.2º
*c* = 10.7369 (4) Å	µ = 0.91 mm^−^^1^
α = 82.047 (1)º	*T* = 273 (2) K
β = 76.289 (1)º	Block, purple
γ = 64.941 (1)º	0.20 × 0.15 × 0.12 mm
*V* = 989.32 (7) Å^3^	

### Data collection

**Table:**

Bruker SMART CCD area-detector diffractometer	3261 reflections with *I* > 2σ(*I*)
Radiation source: fine-focus sealed tube	*R*_int_ = 0.017
Monochromator: graphite	θ_max_ = 25.1º
φ and ω scans	θ_min_ = 2.0º
Absorption correction: multi-scan(SADABS; Sheldrick, 1996)	*h* = −9→11
*T*_min_ = 0.840, *T*_max_ = 0.899	*k* = −12→12
11346 measured reflections	*l* = −12→12
3486 independent reflections	

### Refinement

**Table:**

Refinement on *F*^2^	Secondary atom site location: difference Fourier map
Least-squares matrix: full	Hydrogen site location: inferred from neighbouring sites
*R*[*F*^2^ > 2σ(*F*^2^)] = 0.028	H atoms treated by a mixture of independent and constrained refinement
*wR*(*F*^2^) = 0.094	*w* = 1/[σ^2^(*F*_o_^2^) + (0.073*P*)^2^ + 0.17*P*] where *P* = (*F*_o_^2^ + 2*F*_c_^2^)/3
*S* = 1.00	(Δ/σ)_max_ = 0.001
3486 reflections	Δρ_max_ = 0.28 e Å^−^^3^
281 parameters	Δρ_min_ = −0.36 e Å^−^^3^
Primary atom site location: structure-invariant direct methods	Extinction correction: none

### Special details

**Table:**

Geometry. All e.s.d.'s (except the e.s.d. in the dihedral angle between two l.s. planes) are estimated using the full covariance matrix. The cell e.s.d.'s are taken into account individually in the estimation of e.s.d.'s in distances, angles and torsion angles; correlations between e.s.d.'s in cell parameters are only used when they are defined by crystal symmetry. An approximate (isotropic) treatment of cell e.s.d.'s is used for estimating e.s.d.'s involving l.s. planes.
Refinement. Refinement of *F*^2^ against ALL reflections. The weighted *R*-factor *wR* and goodness of fit *S* are based on *F*^2^, conventional *R*-factors *R* are based on *F*, with *F* set to zero for negative *F*^2^. The threshold expression of *F*^2^ > σ(*F*^2^) is used only for calculating *R*-factors(gt) *etc*. and is not relevant to the choice of reflections for refinement. *R*-factors based on *F*^2^ are statistically about twice as large as those based on *F*, and *R*- factors based on ALL data will be even larger.

### Fractional atomic coordinates and isotropic or equivalent isotropic displacement parameters (Å^2^)

**Table:**

	*x*	*y*	*z*	*U*_iso_*/*U*_eq_	
Co1	0.21615 (2)	0.26369 (2)	0.46144 (2)	0.03237 (12)	
O1	0.36577 (14)	0.20861 (13)	0.56554 (12)	0.0381 (3)	
O2	0.46440 (17)	0.29619 (16)	0.67761 (14)	0.0553 (4)	
O3	0.05933 (15)	0.33761 (13)	0.60596 (13)	0.0418 (3)	
O4	0.06935 (14)	0.32742 (13)	0.35430 (13)	0.0421 (3)	
O5	0.4828 (3)	0.4912 (3)	0.2562 (2)	0.1083 (9)	
H5	0.4933	0.5573	0.2770	0.162*	
N1	0.18775 (16)	0.09298 (15)	0.50938 (14)	0.0348 (3)	
N2	0.36569 (17)	0.19377 (15)	0.30857 (14)	0.0349 (3)	
N3	0.23374 (19)	0.43960 (16)	0.43835 (17)	0.0397 (4)	
C1	0.0225 (2)	0.23999 (19)	0.67578 (17)	0.0391 (4)	
C2	−0.0769 (2)	0.2598 (2)	0.7924 (2)	0.0539 (5)	
H2	−0.1231	0.3465	0.8279	0.065*	
C3	−0.1088 (3)	0.1483 (3)	0.8583 (2)	0.0683 (7)	
H3	−0.1735	0.1626	0.9386	0.082*	
C4	−0.0487 (3)	0.0206 (3)	0.8091 (2)	0.0656 (6)	
H4	−0.0744	−0.0498	0.8543	0.079*	
C5	0.0530 (2)	−0.0043 (2)	0.6888 (2)	0.0460 (5)	
C6	0.1205 (2)	−0.1297 (2)	0.6232 (2)	0.0528 (5)	
H6	0.0999	−0.2057	0.6600	0.063*	
C7	0.2144 (2)	−0.1391 (2)	0.5076 (2)	0.0504 (5)	
H7	0.2567	−0.2210	0.4642	0.060*	
C8	0.2488 (2)	−0.02614 (18)	0.45221 (19)	0.0413 (4)	
H8	0.3162	−0.0354	0.3735	0.050*	
C9	0.08930 (19)	0.10617 (18)	0.62504 (17)	0.0364 (4)	
C10	0.1360 (2)	0.30635 (18)	0.23300 (19)	0.0422 (4)	
C11	0.0622 (3)	0.3473 (2)	0.1301 (2)	0.0590 (6)	
H11	−0.0435	0.3965	0.1439	0.071*	
C12	0.1463 (4)	0.3150 (3)	0.0052 (2)	0.0690 (7)	
H12	0.0939	0.3445	−0.0622	0.083*	
C13	0.3015 (4)	0.2421 (3)	−0.0224 (2)	0.0657 (7)	
H13	0.3530	0.2230	−0.1068	0.079*	
C14	0.3826 (3)	0.1964 (2)	0.07908 (19)	0.0500 (5)	
C15	0.5417 (3)	0.1158 (2)	0.0668 (2)	0.0576 (5)	
H15	0.6026	0.0903	−0.0139	0.069*	
C16	0.6059 (2)	0.0755 (2)	0.1726 (2)	0.0558 (5)	
H16	0.7101	0.0205	0.1645	0.067*	
C17	0.5149 (2)	0.1171 (2)	0.29365 (19)	0.0438 (4)	
H17	0.5602	0.0904	0.3653	0.053*	
C18	0.2987 (2)	0.23139 (18)	0.20409 (18)	0.0393 (4)	
C19	0.3819 (2)	0.31043 (19)	0.60261 (16)	0.0392 (4)	
C20	0.2899 (2)	0.45316 (19)	0.5487 (2)	0.0452 (4)	
H20A	0.2040	0.5039	0.6144	0.054*	
H20B	0.3538	0.5047	0.5221	0.054*	
C21	0.5849 (6)	0.4394 (5)	0.1517 (4)	0.1377 (19)	
H21A	0.5343	0.4321	0.0885	0.207*	
H21B	0.6579	0.3490	0.1722	0.207*	
H21C	0.6367	0.4993	0.1183	0.207*	
H22	0.143 (3)	0.502 (3)	0.436 (2)	0.060 (7)*	
H21	0.295 (3)	0.444 (2)	0.369 (2)	0.048 (6)*	

### Atomic displacement parameters (Å^2^)

**Table:**

	*U*^11^	*U*^22^	*U*^33^	*U*^12^	*U*^13^	*U*^23^
Co1	0.02705 (16)	0.02604 (16)	0.04380 (17)	−0.01193 (11)	−0.00651 (10)	0.00211 (10)
O1	0.0359 (7)	0.0324 (7)	0.0464 (7)	−0.0132 (5)	−0.0107 (5)	−0.0006 (5)
O2	0.0585 (9)	0.0602 (9)	0.0529 (8)	−0.0229 (7)	−0.0185 (7)	−0.0116 (7)
O3	0.0365 (7)	0.0317 (6)	0.0529 (7)	−0.0144 (5)	0.0006 (5)	−0.0027 (5)
O4	0.0315 (6)	0.0366 (7)	0.0594 (8)	−0.0142 (5)	−0.0147 (6)	0.0056 (6)
O5	0.1061 (17)	0.1167 (19)	0.1255 (19)	−0.0846 (16)	0.0328 (15)	−0.0453 (15)
N1	0.0294 (7)	0.0310 (7)	0.0468 (8)	−0.0139 (6)	−0.0126 (6)	0.0036 (6)
N2	0.0330 (8)	0.0318 (7)	0.0431 (8)	−0.0165 (6)	−0.0089 (6)	0.0020 (6)
N3	0.0316 (8)	0.0305 (8)	0.0567 (10)	−0.0144 (7)	−0.0078 (7)	0.0035 (7)
C1	0.0321 (9)	0.0415 (10)	0.0468 (10)	−0.0187 (7)	−0.0087 (7)	0.0023 (8)
C2	0.0489 (12)	0.0610 (13)	0.0523 (11)	−0.0273 (10)	0.0013 (9)	−0.0072 (10)
C3	0.0685 (15)	0.0868 (18)	0.0532 (12)	−0.0460 (14)	0.0061 (11)	0.0020 (12)
C4	0.0710 (15)	0.0741 (16)	0.0641 (14)	−0.0506 (13)	−0.0070 (12)	0.0173 (12)
C5	0.0414 (10)	0.0463 (11)	0.0589 (11)	−0.0272 (9)	−0.0163 (9)	0.0131 (9)
C6	0.0506 (11)	0.0379 (10)	0.0813 (15)	−0.0285 (9)	−0.0242 (11)	0.0152 (9)
C7	0.0458 (11)	0.0330 (9)	0.0788 (14)	−0.0189 (8)	−0.0193 (10)	−0.0011 (9)
C8	0.0377 (9)	0.0336 (9)	0.0547 (11)	−0.0146 (7)	−0.0135 (8)	−0.0003 (8)
C9	0.0310 (9)	0.0374 (9)	0.0461 (9)	−0.0185 (7)	−0.0135 (7)	0.0070 (7)
C10	0.0461 (10)	0.0310 (9)	0.0590 (12)	−0.0226 (8)	−0.0218 (9)	0.0108 (8)
C11	0.0656 (14)	0.0484 (12)	0.0783 (15)	−0.0302 (11)	−0.0415 (12)	0.0196 (11)
C12	0.101 (2)	0.0613 (15)	0.0661 (15)	−0.0430 (15)	−0.0491 (15)	0.0208 (12)
C13	0.104 (2)	0.0626 (14)	0.0471 (12)	−0.0481 (15)	−0.0239 (12)	0.0099 (10)
C14	0.0691 (14)	0.0482 (12)	0.0451 (10)	−0.0369 (11)	−0.0115 (10)	0.0026 (9)
C15	0.0618 (14)	0.0653 (14)	0.0516 (12)	−0.0374 (11)	0.0071 (10)	−0.0148 (10)
C16	0.0408 (11)	0.0627 (13)	0.0630 (13)	−0.0224 (10)	0.0013 (9)	−0.0159 (10)
C17	0.0340 (9)	0.0455 (10)	0.0523 (10)	−0.0160 (8)	−0.0078 (8)	−0.0051 (8)
C18	0.0472 (10)	0.0330 (9)	0.0463 (10)	−0.0241 (8)	−0.0143 (8)	0.0062 (7)
C19	0.0337 (9)	0.0423 (10)	0.0383 (9)	−0.0145 (8)	0.0010 (7)	−0.0084 (7)
C20	0.0412 (10)	0.0362 (9)	0.0614 (12)	−0.0182 (8)	−0.0087 (9)	−0.0070 (8)
C21	0.197 (5)	0.142 (4)	0.116 (3)	−0.129 (4)	0.030 (3)	−0.047 (3)

### Geometric parameters (Å, °)

**Table:**

Co1—O1	1.9045 (12)	C5—C6	1.417 (3)
Co1—O3	1.8926 (13)	C6—C7	1.348 (3)
Co1—O4	1.9002 (13)	C6—H6	0.9300
Co1—N1	1.9373 (14)	C7—C8	1.403 (3)
Co1—N2	1.9179 (15)	C7—H7	0.9300
Co1—N3	1.9309 (15)	C8—H8	0.9300
O1—C19	1.287 (2)	C10—C11	1.384 (3)
O2—C19	1.225 (2)	C10—C18	1.431 (3)
O3—C1	1.323 (2)	C11—C12	1.400 (4)
O4—C10	1.314 (2)	C11—H11	0.9300
O5—C21	1.319 (4)	C12—C13	1.367 (4)
O5—H5	0.8200	C12—H12	0.9300
N1—C8	1.320 (2)	C13—C14	1.413 (3)
N1—C9	1.365 (2)	C13—H13	0.9300
N2—C17	1.326 (2)	C14—C18	1.405 (3)
N2—C18	1.362 (2)	C14—C15	1.413 (3)
N3—C20	1.468 (3)	C15—C16	1.361 (3)
N3—H22	0.86 (3)	C15—H15	0.9300
N3—H21	0.85 (2)	C16—C17	1.399 (3)
C1—C2	1.374 (3)	C16—H16	0.9300
C1—C9	1.419 (3)	C17—H17	0.9300
C2—C3	1.410 (3)	C19—C20	1.518 (3)
C2—H2	0.9300	C20—H20A	0.9700
C3—C4	1.359 (4)	C20—H20B	0.9700
C3—H3	0.9300	C21—H21A	0.9600
C4—C5	1.414 (3)	C21—H21B	0.9600
C4—H4	0.9300	C21—H21C	0.9600
C5—C9	1.414 (2)		
			
O3—Co1—O4	90.60 (6)	C6—C7—H7	119.8
O3—Co1—O1	89.93 (6)	C8—C7—H7	119.8
O4—Co1—O1	176.81 (5)	N1—C8—C7	121.45 (18)
O3—Co1—N2	176.47 (5)	N1—C8—H8	119.3
O4—Co1—N2	85.88 (6)	C7—C8—H8	119.3
O1—Co1—N2	93.56 (6)	N1—C9—C5	122.64 (17)
O3—Co1—N3	87.07 (7)	N1—C9—C1	115.27 (15)
O4—Co1—N3	91.14 (6)	C5—C9—C1	122.07 (17)
O1—Co1—N3	85.75 (6)	O4—C10—C11	125.8 (2)
N2—Co1—N3	92.71 (7)	O4—C10—C18	117.56 (16)
O3—Co1—N1	85.82 (6)	C11—C10—C18	116.6 (2)
O4—Co1—N1	92.39 (6)	C10—C11—C12	120.2 (2)
O1—Co1—N1	90.79 (6)	C10—C11—H11	119.9
N2—Co1—N1	94.60 (6)	C12—C11—H11	119.9
N3—Co1—N1	172.09 (7)	C13—C12—C11	123.2 (2)
C19—O1—Co1	113.96 (11)	C13—C12—H12	118.4
C1—O3—Co1	111.40 (11)	C11—C12—H12	118.4
C10—O4—Co1	111.26 (11)	C12—C13—C14	119.0 (2)
C21—O5—H5	109.5	C12—C13—H13	120.5
C8—N1—C9	119.18 (15)	C14—C13—H13	120.5
C8—N1—Co1	131.33 (13)	C18—C14—C13	117.7 (2)
C9—N1—Co1	109.46 (12)	C18—C14—C15	116.45 (19)
C17—N2—C18	119.37 (17)	C13—C14—C15	125.8 (2)
C17—N2—Co1	129.99 (13)	C16—C15—C14	120.2 (2)
C18—N2—Co1	110.63 (12)	C16—C15—H15	119.9
C20—N3—Co1	107.24 (11)	C14—C15—H15	119.9
C20—N3—H22	111.7 (16)	C15—C16—C17	119.9 (2)
Co1—N3—H22	106.0 (17)	C15—C16—H16	120.1
C20—N3—H21	110.4 (15)	C17—C16—H16	120.1
Co1—N3—H21	111.4 (15)	N2—C17—C16	121.50 (18)
H22—N3—H21	110 (2)	N2—C17—H17	119.3
O3—C1—C2	124.64 (18)	C16—C17—H17	119.3
O3—C1—C9	117.25 (15)	N2—C18—C14	122.54 (18)
C2—C1—C9	118.10 (17)	N2—C18—C10	114.32 (17)
C1—C2—C3	119.9 (2)	C14—C18—C10	123.13 (18)
C1—C2—H2	120.1	O2—C19—O1	123.35 (17)
C3—C2—H2	120.1	O2—C19—C20	120.82 (17)
C4—C3—C2	122.5 (2)	O1—C19—C20	115.83 (15)
C4—C3—H3	118.7	N3—C20—C19	109.90 (15)
C2—C3—H3	118.7	N3—C20—H20A	109.7
C3—C4—C5	119.62 (19)	C19—C20—H20A	109.7
C3—C4—H4	120.2	N3—C20—H20B	109.7
C5—C4—H4	120.2	C19—C20—H20B	109.7
C4—C5—C9	117.7 (2)	H20A—C20—H20B	108.2
C4—C5—C6	126.22 (19)	O5—C21—H21A	109.5
C9—C5—C6	116.04 (18)	O5—C21—H21B	109.5
C7—C6—C5	120.19 (17)	H21A—C21—H21B	109.5
C7—C6—H6	119.9	O5—C21—H21C	109.5
C5—C6—H6	119.9	H21A—C21—H21C	109.5
C6—C7—C8	120.45 (19)	H21B—C21—H21C	109.5

### Hydrogen-bond geometry (Å, °)

**Table:**

*D*—H···*A*	*D*—H	H···*A*	*D*···*A*	*D*—H···*A*
O5—H5···O2^i^	0.82	1.92	2.743 (3)	175
N3—H21···O5	0.85 (2)	2.17 (3)	2.950 (3)	153 (2)
N3—H22···O3^ii^	0.86 (3)	2.10 (3)	2.952 (2)	169 (2)

Symmetry codes: (i) −*x*+1, −*y*+1, −*z*+1; (ii) −*x*, −*y*+1, −*z*+1.
